# The predictors of fungal infections after liver transplantation and the influence of fungal infections on outcomes

**DOI:** 10.1007/s10238-024-01419-8

**Published:** 2024-07-03

**Authors:** Juan Jiang, Peng Peng, Qiquan Wan

**Affiliations:** 1 Department of Nephrology, The Third Xiangya Hospital of Central South University, Changsha, 410013 China; 2 Clinical Laboratory Medicine Center, Xiangya Hospital Zhuzhou of Central South University, Zhuzhou, 421007 China; 3 Department of Transplant Surgery, The Third Xiangya Hospital of Central South University, Changsha, 410013 China; 4 Engineering and Technology Research Center for Transplantation Medicine of National Health Commission, The Third Xiangya Hospital of Central South University, Changsha, 410013 China

**Keywords:** Liver transplantation, Fungal infections, Risk factors, Prognosis

## Abstract

The primary objective of this study was to assess the incidence, timing, risk factors of fungal infections (FIs) within 3 months after liver transplantation (LT). The secondary objective was to evaluate the impact of FIs on outcomes. Four hundred and ten patients undergoing LT from January 2015 until January 2023 in a tertiary university hospital were included in the present retrospective cohort study to investigate the risk factors of FIs and to assess the impacts of FIs on the prognosis of LT recipients using logistic regression. The incidence of FIs was 12.4% (51/410), and median time from LT to the onset of FIs was 3 days. By univariate analysis, advanced recipient age, prolonged hospital stay prior to LT, high Model for End Stage Liver Disease (MELD) score, use of broad-spectrum antibiotics, and elevated white blood cell (WBC) count, increased operating time, massive blood loss and red blood cell transfusion, elevated alanine aminotransferase on day 1 and creatinine on day 3 after LT, prolonged duration of urethral catheter, prophylactic antifungal therapy, the need for mechanical ventilation and renal replacement therapy were identified as factors of increased post-LT FIs risk. Multivariate logistic regression analysis identified that recipient age ≥ 55 years[OR = 2.669, 95%CI: 1.292–5.513, *P* = 0.008], MELD score at LT ≥ 22[OR = 2.747, 95%CI: 1.274–5.922, *P* = 0.010], pre-LT WBC count ≥ 10 × 10^9^/L[OR = 2.522, 95%CI: 1.117–5.692, *P* = 0.026], intraoperative blood loss ≥ 3000 ml [OR = 2.691, 95%CI: 1.262–5.738, *P* = 0.010], post-LT duration of urethral catheter > 4 d [OR = 3.202, 95%CI: 1.553–6.602, *P* = 0.002], and post-LT renal replacement therapy [OR = 5.768, 95%CI: 1.822–18.263, *P* = 0.003] were independently associated with the development of post-LT FIs. Post-LT prophylactic antifungal therapy ≥ 3 days was associated with a lower risk of the development of FIs [OR = 0.157, 95%CI: 0.073–0.340, *P* < 0.001]. As for clinical outcomes, FIs had a negative impact on intensive care unit (ICU) length of stay ≥ 7 days than those without FIs [OR = 3.027, 95% CI: 1.558–5.878, *P* = 0.001] but had no impact on hospital length of stay and 1-month all-cause mortality after LT. FIs are frequent complications after LT and the interval between the onset of FIs and LT was short. Risk factors for post-LT FIs included high MELD score at LT, advanced recipient age, pre-LT WBC count, massive intraoperative blood loss, prolonged post-LT duration of urethral catheter, and the need for post-LT renal replacement therapy. However, post-LT prophylactic antifungal therapy was independently associated with the reduction in the risk of FIs. FIs had a significant negative impact on ICU length of stay.

## Core tip

Despite advances in technology of liver transplantation, fungal infections remain challenging. Timely prevention of IF is critical. Many risk factors play a crucial role in the occurrence of FIs after LT and determine the prognosis of recipients. This study is one of the comprehensive studies to examine the role of FI in the prognosis of liver transplant recipients and the risk factors for all FIs after liver transplantation and to demonstrate that the risk factors are associated with preoperative, intraoperative and postoperative variables. the identification of these risk factors for FIs provides a basis for the prevention of FIs, which in turn improves the prognosis of LT recipients

## Introduction

Liver transplantation (LT) is an effective treatment for end-stage liver diseases and certain malignancies [1]. LT recipients have a significant risk of fungal infections (FIs) which are outcome-determining complications in the early period after LT [2]. The overall incidence of invasive FIs in LT recipients is up to 42%, with an related mortality rate up to 80% [3–7]. LT recipients with invasive FIs were nearly five times more likely to die than those without [1]. The most common fungal pathogens (53%–93%) are *Candida* spp., followed by *Aspergillus* spp.(1.4%-19%) and *Cryptococcus* spp. (0.5%-8%) [8–10]. Approximately 34% and 46% of invasive *Candida* infections occur within one and three months after LT, respectively, earlier than *Aspergillus* infections do [1, 8].

Multiple previous studies stated many risk factors for invasive FIs, including hospitalization or antiinfective treatment before transplantation, an elevated Model for End Stage Liver Disease (MELD) score and serum creatinine, preexisting end-stage renal disease, fungal colonization prior to LT, live donor, donor derived infections, split-LT, prolonged operation duration, massive transfusion, Roux-en-y choledocho-jejunostomy, postoperative bacterial infection, acute kidney injury, the need for renal replacement therapy, early re-operation, CMV viremia, and biliary leakage, but the consensus is still missing to date [8, 11–19].

Although some studies examining the effects of invasive FIs on the prognosis of LT recipients as well as the risk factors for invasive FIs, only a few studies examined these issues in all FIs including invasive and noninvasive FIs. Our present work is one of those studies in an integrative manner to examine the role of all FIs in prognosis of LT recipients as well as the risk factors for all FIs after LT, and demonstrated the risk factors were pre-, intra- and, post-operative variables-related, and LT recipients with FIs had a longer intensive care unit (ICU) stay than those without. We identified these risk factors for FIs to provide a basis of preventing FIs and then to improve the prognosis of LT recipients.

## Materials and methods

### General information

We conducted a retrospective cohort study including all adult patients who received a LT between January 2015 and January 2023 at the Third Xiangya Hospital, a 1915-bed tertiary care hospital affiliated to Central South University performing liver transplants since 2001. Eventually, 410 LT recipients of grafts from donation after citizens’ death were eligible in this single-center retrospective cohort study. All liver grafts were from donation after brain death with the exception of one from donation after circulatory death. This study received approval by the Ethics Committee of the Third Xiangya Hospital, and written patient consent was waived because of the retrospective nature of the study (no.23776).

### Inclusion and exclusion criteria

Inclusion criteria were adult (aged 18 years old and older) LT recipients. We excluded LT recipients who were younger than 18 years old, presented infections within two weeks before LT, or died during the peri-operative period for anesthesia accidents or surgical complications.

### Definitions and assessment of FIs

Infection was confirmed based on the criteria of the Centers for Disease Control and Prevention/National Healthcare Safety Network (CDC/NHSN) [20]. FIs were defined according to updated EORTC/MSGERC consensus definitions for non-lung solid organ transplant recipients [21]. Invasive FIs were defined as clinical manifestations and fungal isolation with presence of organ damage by radiology, bronchoscopy or biopsy in the absence of other causes. In noninvasive FIs organ damage was not present [22]. Re-operation implicated in retransplantation or post-LT laparotomy. Acute rejection was T cell- or antibody- mediated and was biopsy-proven.

### Treatment strategy

All patients were received a modified piggyback LT. A T-tube was not placed for biliary drainage except for 6 (1.5%) patients. All LT recipients were exposed to antibiotic prophylaxis for 3–5 days with the same third-generation cephalosporin or carbapenem used prior to or during LT according to the protocol of our center. Teicoplanin or other antibiotics against bacterial infections were prescribed with a treatment duration of 5–7 days when needed. The antifungal prophylaxis using caspofungin or another echinocandin for duration of 7–14 days was reserved for LT recipients having at least 2 predefined perioperative risk factors (such as retransplantation, renal dysfunction or fungi colonization prior to LT, choledochojejuno-stomy, massive transfusion, prolonged operating time, and so on), which was recommended by substantial studies [23–25].

An intraoperative 500 mg methylprednisolone bolus was administered intravenously as an initial treatment, with subsequent tapering over one week, and basiliximab induction was additionally combined in 217 (52.9%) LT recipients. Maintenance immunosuppressive therapy consisted of a double drug regimen including a calcineurin inhibitor (tacrolimus or ciclosporin A) and corticosteroid with or without an antimetabolite (Mycophenolate mofetil or enteric-coated mycophenolate sodium). Anti-thymocyte globulin was prescribed when needed. All patients after LT were closely monitored in the ICU for 3–7 days.

### The content and acquisition methods of the data

The electronic medical records were reviewed for all LT recipients over 8 years. We also obtained patients’ information through regular outpatient follow-up. We collected general peri-operative conditions of the patients and all prespecified data possibly related to FIs, and postoperative outcomes (mortality, ICU and hospital length of stay). All LT patients were followed up for 3 months postoperatively for their microbiological data. Samples, including blood, urine, sputum, or abdominal drainage fluid, were routinely subject to bacterial culture once a day for 5–7 days following LT. Thereafter, if infection is suspected, samples were collected for culture within the 3 months after LT. When an infection was suspected, chest film, computerized tomography, blood routine, C-reactive protein, or procalcitonin, one or more of which will also be tested. All infections were distinguished from contamination.

### Statistical analyses

All statistical analyses were performed using the SPSS software, version 26.0 statistical (SPSS, Inc., Chicago, IL). Continuous variables were presented as mean ± standard deviation if normally distributed or the median and interquartile range when non-normally distributed. Categorical data were expressed as absolute numbers and their percent. Pearson’s chi-square test or Fisher's exact test was used to test categorical variables, as appropriate. The significant risk factors identified in univariate analysis (*P* < 0.05) were included in the final multivariate analysis where binary logistic regression on the basis of forward stepwise logistic regression was used to determine risk factors with an odds ratio (OR) value along with a 95% confidence interval (CI). *P* < 0.05 was determined to represent statistical significance, and statistical assessments were two-tailed.

## Results

### General characteristics and prognosis of LT recipients

We retrospectively reviewed all patients who received a LT of graft from a deceased donor during the study period. Of 414 patients screened, 4 patients were excluded; 1 patient each died of massive blood loss during the operation and primary graft non-function, and 2 patients were younger than 18 years old. A total of 410 patients were eventually included in this study with median MELD score of 23. The mean age was 47.3 years old (± 10.6), and males accounted for 82.4% of the population. The majority of the patients underwent LT due to hepatitis virus-related cirrhosis/necrosis/tumor (n = 308). The remaining cases involved alcoholic liver disease (n = 31), mixed cirrhosis (n = 19), autoimmune hepatitis (n = 15), primary biliary cirrhosis (n = 11), cryptogenic cirrhosis (n = 9), Budd-Chiari syndrome (n = 5), hepatolenticular degeneration (n = 3), transplanted liver failure (n = 3), drug-induced liver injury (n = 2), polycystic liver (n = 2), and familial hereditary amyloidosis (n = 2). Infections within two months before LT accounted for 40.2% (165/410) of all patients with 45 (35.4%) suffering from pulmonary infections and 13 (3.2%) multiple sites infections, all of whom had pulmonary infections.

It was 0.8 mg/dL, 34.5 g/L, 5.3 × 10^9^/L, 0.8 × 10^9^/L, and 72.0 × 10^9^/L for the median pre-LT creatinine, albumin, WBC count, lymphocyte count, and platelet count, respectively.

The median surgery time, blood loss and RBC transfusion were 378.5 min, 3000.0 ml and 12.0 units, respectively.

A total of 51 (12.4%) patients were infected with 55 strains of fungus within 3 months. Median time from transplantation to FIs was 3 days. Among these 55 strains of fungus, *Candida* spp. accounted for 84.3% of the fungi isolated and *Aspergillus fumigatus* a further 7.8%. Invasive FIs accounted for 45.1% (23/51) of all FIs. Of 410 LT patients involved, 385 (93.9%) were prescribed a carbapenem and the remaining patients (6.1%; 25/410) used a third-generation cephalosporin. After LT, 18 (4.4%) patients were treated with anti-thymocyte immunoglobulin therapy, and 399 (97.3%) with tacrolimus. The median alanine aminotransferase (ALT) and albumin on day 1 and median creatinine on day 3 after LT were 696.0 U/L, 37.2 g/L, and 0.9 mg/dL, respectively. A total 95 patients required mechanical ventilation, 19 needed renal replacement therapy, and 67 arose acute rejection after LT. Moreover, 4.1% (17/410) of cases received re-operation. The median postoperative ICU length of stay was 6.0 days. We observed 1-month mortality of 6.6% (27/410). The baseline demographic, clinical, and laboratory characteristics were described in Table 1.

**Table 1:** Demographic, laboratory, and clinical variables of 410 LT recipients

Characteristics	Value
Recipient age, mean years ± SD	47.3 ± 10.6
Recipient gender, no. of male (%)	338(82.4)
Recipient BMI, median (IQR), kg/m^2^	22.8(20.7–25.1)
Hospital stay prior to LT, median (IQR), days	10(1–22.3)
MELD score at LT, median (IQR)	23(15–30)
Infection within 2 months prior to LT, n (%)	165(40.2)
Pulmonary infection	145(35.4)
Abdominal/biliary infection	6(1.5)
Urinary tract infection	1(0.2)
Multiple site infection^1^	13(3.2)
Pre-LT use of broad-spectrum antibiotics	167(40.7)
Underlying liver diseases, n (%)	410(100)
Viral cirrhosis/necrosis/tumor	308(75.1)
Alcoholic cirrhosis	31(7.6)
Autoimmune hepatitis	15(3.7)
Primary biliary cirrhosis	11(2.7)
Mixed cirrhosis	19(4.6)
Others^2^	26(6.3)
Pre-LT type 2 diabetes, n (%)	48(11.7)
Pre-LT creatinine, median (IQR), mg/dL	0.8(0.7–1)
Pre-LT WBC count, median (IQR), × 10^9^/L	5.3(3.5–8.1)
Pre-LT lymphocyte count, median (IQR), × 10^9^/L	0.8(0.5–1.2)
Pre-LT platelet count, median (IQR), × 10^9^/L	72(43.8–106.5)
Pre-LT albumin level, median (IQR), g/L	34.5(30.9–38.1)
Donor age, mean years ± SD	42.1 ± 13
Steatosis ≥ 30%, n (%)	42(10.2)
Cold ischemia time, mean h ± SD	6.1 ± 1.7
Duration of surgery, median (IQR), min	378.5(333–425)
Intraoperative bleeding, median (IQR), mL	3000(2000–5000)
Intraoperative RBC transfusion, median (IQR), units	12(8–18)
Post-LT FIs, n (%)	51(12.4)
*Candida albicans*	20(4.9)
*Candida parapsilosis*	8(2.0)
*Candida glabrata*	7(1.7)
*Candida krusei*	2(0.5)
*Candida tropicalis*	2(0.5)
*Candida guilliermondii*	1(0.2)
*Aspergillus fumigatus*	4(1.0)
*Trichosporon asahii*	1(0.2)
*Pneumocystis Jiroveci*	1(0.2)
*Lichtheimia ramosa*	1(0.2)
Mixed fungal infection^3^	4(1.0)
Invasive FIs, n (%)	23(5.6)
Post-LT prophylactic antifungal therapy ≥ 3 d, n (%)	222(54.1)
Post-LT prophylaxis with third-generation cephalosporins, n (%)	25(6.1)
Post-LT prophylaxis with carbapenems, n (%)	385(93.9)
Post-LT immunosuppressant treatment, n (%)	410(100)
Tacrolimus	399(97.3)
Ciclosporin A	5(1.2)
Mycophenolate mofetil/enteric-coated mycophenolate sodium	279(68)
Sirolimus	5(1.2)
Glucocorticoid	410(100)
Basiliximab	217(52.9)
Anti-thymocyte globulin	18(4.4)
ALT on day 1 after LT, median (IQR), U/L	696(383–1300)
Creatinine on day 3 after LT, median (IQR), mg/dL	0.9(0.7–1.4)
Albumin level on day 1 after LT, median (IQR), g/L	37.2(33.9–40.7)
Post-LT duration of urethral catheter, median (IQR), days	3(2–5)
Post-LT mechanical ventilation, n (%)	95(23.2)
Reoperation, n (%)	17(4.1)
Post-LT bacterial infection, n (%)	106(25.9)
Acute rejection, n (%)	67(16.3)
Post-LT renal replacement therapy, n (%)	19(4.6)
ICU stay after LT, median (IQR), days	6(5–7)
Hospitalization stay after LT, median (IQR), days	26(21–30)
All-cause mortality within 1 month after LT, n (%)	27(6.6)

*ALT* alanine aminotransferase, *BMI* body mass index, *ICU* intensive care unit, *FIs* fungal infections, *IQR* interquartile range, *LT* liver transplantation, *MELD* Model for end-stage liver disease, *RBC* red blood cell, *SD* standard deviation, *WBC* white blood cell

^1^There were 9 cases of pulmonary and abdominal/bile duct infections, 1 case of pulmonary and urinary tract infections, 1 case of pulmonary and bloodstream infections, 1 case of pulmonary and intracranial infections, and 1 case each of pulmonary, abdominal and bloodstream infections

^2^There were 9 cases of cryptogenic cirrhosis, 5 cases of Budd-Chiari syndrome, 3 cases each of hepatolenticular degeneration and transplant liver failure, 2 cases each of drug-induced liver injury, polycystic liver, and familial hereditary amyloidosis

^3^There were 2 cases of *Candida albicans* and *Candida glabrata* infections, 1 case of *Candida albicans* and *Candida tropicalis* infections, 1 case of *Saccharomyces cerevisiae* and *Cryptococcus neoformans* infections

### Analysis of the risk factors for FIs after LT

A comparison of patients with and without FIs was performed. By univariate logistic regression, recipient age ≥ 55 years (*P* = 0.004), MELD score at LT ≥ 22 (*P* = 0.005), pre-LT hospitalization ≥ 7 days (*P* = 0.007), pre-LT use of broad-spectrum antibiotics ≥ 3 days (*P* = 0.012), pre-LT WBC count ≥ 10 × 10^9^/L (*P* = 0.005), duration of surgery ≥ 450 min (*P* = 0.017), intraoperative blood loss ≥ 3000 ml (*P* = 0.004), intraoperative RBC transfusion ≥ 12 U (*P* = 0.013), ALT on day 1 after LT ≥ 1000 U/L (*P* = 0.039), creatinine on day 3 after LT ≥ 2 mg/dL (*P* = 0.023), post-LT duration of urethral catheter ≥ 4 days (*P* < 0.001), post-LT prophylactic antifungal therapy ≥ 3 days (*P* < 0.001), and the need for post-LT mechanical ventilation (*P* < 0.001) and renal replacement therapy (*P* < 0.001) were associated with an increased risk for post-LT FIs.

Finally, the multivariate analysis identified recipient age ≥ 55 years [OR = 2.669, 95%CI:1.292–5.513, *P* = 0.008], MELD score at LT ≥ 22 [OR = 2.747, 95%CI:1.274–5.922, *P* = 0.010], pre-LT WBC count ≥ 10 × 10^9^/L [OR = 2.522, 95%CI:1.117–5.692, *P* = 0.026], intraoperative blood loss ≥ 3000 ml [OR = 2.691, 95%CI:1.262–5.738, *P* = 0.010], post-LT duration of urethral catheter > 4 d [OR = 3.202, 95%CI:1.553–6.602, *P* = 0.002], and post-LT renal replacement therapy [OR = 5.768, 95%CI:1.822–18.263, *P* = 0.003] as independent risk factors for post-LT FIs, which were reduced in LT recipients with post-LT prophylactic antifungal therapy ≥ 3 days [OR = 0.157, 95%CI:0.073–0.340, *P* < 0.001]. All data regarding the univariate and multivariate analysis were displayed in Table 2.

**Table 2:** Univariate analysis of risk factors for IFIs in LT recipients

Variables	FIs (51)	No FIs (359)	*P*	OR (95%CI)
Total, *n* (%)				
Univariate analysis				
Male sex, *n* (%)	40(78.4)	298(83)	0.421	
Recipient age ≥ 55 years, *n* (%)	21(41.2)	81(22.6)	0.004	
Recipient BMI ≥ 25	8(15.7)	99(27.6)	0.070	
MELD score at LT ≥ 22, *n* (%)	38(74.5)	193(53.8)	0.005	
Hospital stay prior to LT ≥ 7 days, *n* (%)	39(76.5)	203(56.5)	0.007	
Viral cirrhosis/necrosis/tumor, *n* (%)	38(74.5)	266(74.1)	0.949	
Alcoholic cirrhosis, *n* (%)	5(9.8)	27(7.5)	0.57	
Pre-LT diabetes, *n* (%)	7(13.7)	41(11.4)	0.632	
Pre-LT use of broad-spectrum antibiotics ≥ 3 d	29(56.9)	138(38.4)	0.012	
Pre-LT creatinine ≥ 2 mg/dL, *n* (%)	4(7.8)	25(7)	1.000	
Infection within 2 months prior to LT, *n* (%)	26(51)	136(37.9)	0.073	
Pre-LT WBC count ≥ 10 × 10^9^/L, *n* (%)	14(27.5)	45(12.5)	0.005	
Pre-LT lymphocyte count ≤ 0.5 × 10^9^/L, *n* (%)	11(21.6)	89(24.8)	0.616	
Pre-LT platelet count ≤ 50 × 10^9^/L, *n* (%)	15(29.4)	121(33.7)	0.542	
Pre-LT albumin level < 30 g/L, *n* (%)	5(9.8)	75(20.9)	0.062	
Donor age ≥ 50 years	14(27.5)	121(33.7)	0.374	
Steatosis ≥ 30%, *n* (%)	3(5.9)	39(10.9)	0.272	
Cold ischemia time ≥ 360 min, *n* (%)	25(49.0)	181(50.4)	0.852	
Duration of surgery ≥ 450 min, n (%)	15(29.4)	57(15.9)	0.017	
Intraoperative bleeding ≥ 3000 ml, *n* (%)	39(76.5)	199(55.4)	0.004	
Intraoperative RBC transfusion ≥ 12 U, *n* (%)	36(70.6)	187(52.1)	0.013	
ALT on day 1 after LT ≥ 1000U/L, *n* (%)	23(45.1)	110(30.6)	0.039	
Creatinine on day 3 after LT ≥ 2 mg/dL, *n* (%)	13(25.5)	48(13.4)	0.023	
Albumin level on day 1 after LT < 30 g/L, *n* (%)	1(2)	28(7.8)	0.219	
Post-LT duration of urethral catheter ≥ 4 d	38(74.5)	154(42.9)	< 0.001	
Post-LT prophylactic antifungal therapy ≥ 3 d	14(27.5)	208(57.9)	< 0.001	
Post-LT prophylaxis with carbapenems	51(100)	334(93.0)	0.103	
Post-LT mechanical ventilation, *n* (%)	23(45.1)	72(20.1)	< 0.001	
Reoperation, *n* (%)	4(7.8)	13(3.6)	0.298	
Post-LT bacterial infection, *n* (%)	10(19.6)	96(26.7)	0.276	
Acute rejection, *n* (%)	13(25.5)	54(15)	0.059	
Post-LT renal replacement therapy, *n* (%)	8(15.7)	11(3.1)	< 0.001	
Glucocorticoidse ≥ 1500 mg, *n* (%)	33(64.7)	225(62.7)	0.779	
Basiliximab use ≥ 40 mg, *n* (%)	14(27.5)	147(40.9)	0.065	
Anti-thymocyte globulin use	4(7.8)	14(3.9)	0.357	
Multivariate analysis				
Recipient age ≥ 55 years			0.008	2.669(1.292–5.513)
MELD score at LT ≥ 22			0.010	2.747(1.274–5.922)
Pre-LT WBC count ≥ 10 × 10^9^/L			0.026	2.522(1.117–5.692)
Intraoperative bleeding ≥ 3000 ml			0.010	2.691(1.262–5.738)
Post-LT duration of urethral catheter ≥ 4 d			0.002	3.202(1.553–6.602)
Post-LT renal replacement therapy			0.003	5.768(1.822–18.263)
Post-LT prophylactic antifungal therapy ≥ 3 d			< 0.001	0.157(0.073–0.340)

*ALT* alanine aminotransferase, *BMI* body mass index, *IFIs* invasive fungal infections, *LT* liver transplantation, *MELD* Model for end-stage liver disease, *RBC* red blood cell, *WBC* white blood cell

### Prognosis of patients with FIs after LT

A Pearson’s chi-square test was used to access the effects of FIs on prognosis of LT recipients. Of note, patients with FIs were more likely to stay at ICU ≥ 7 days after LT than those without (*P* < 0.001). Patients with FIs had higher 1-month all-cause mortality rates than those without (17.6% versus 5.0%; *P* = 0.001). In contrast, FIs did not show a significant correlation to hospitalization stay after LT ≥ 21 days (*P* = 0.621) when compared to non-FIs (Table 3). All information was described in Table 3.

**Table 3:** Postoperative outcome for patients with/without FIs following LT

Variables	FIs (51)	No FIs(359)	*χ*^*2*^	*P* value
ICU stay after LT ≥ 7 days, n (%)	34(66.7)	118(32.9)	21.865	< 0.001
Hospitalization stay after LT ≥ 21 days, n (%)	40(78.4)	292(81.3)	0.245	0.621
All-cause mortality within 1 month after LT, n (%)	9(17.6)	18(5.0)	11.585	0.001

*ICU* intensive care unit, *FIs* fungal infections, *LT* liver transplantation

A univariate in conjunction with multivariate analysis of those potential risk factors for mortality was performed to decide if FIs were one of those independent risk factors for 1-month all-cause mortality. Recipient age ≥ 55 years [OR = 3.056, 95%CI:1.168–7.994, *P* = 0.023], creatinine on day 3 after LT ≥ 2 mg/dL [OR = 9.154, 95%CI:3.451–24.280, *P* < 0.001], post-LT bacterial infection [OR = 7.095, 95%CI:2.553–19.714, *P* < 0.001], and post-LT mechanical ventilation [OR = 5.742, 95%CI:2.176–15.151, *P* < 0.001], not FIs, were verified increased risk factors for 1-month mortality after LT. All information was shown in Table 4.

**Table 4:** Univariate and multivariate Logistic regression analysis of risk factors for all-cause mortality within 1 month after LT

Variables	Death (27)	Survival (383)		*P*	OR (95% CI)
*Total, n (%)*					
Univariate analysis					
Male sex, *n* (%)	19(70.4)		319(83.3)	0.088	
Recipient age ≥ 55 years, *n* (%)	14(51.9)		88(23.0)	0.001	
Recipient BMI ≥ 25	3(11.1)		104(27.2)	0.067	
Viral cirrhosis/necrosis/tumor, *n* (%)	21(77.8)		283(73.9)	0.656	
Alcoholic cirrhosis, *n* (%)	1(3.7)		31(8.1)	0.652	
Pre-LT diabetes, *n* (%)	3(11.1)		45(11.7)	1.000	
Hospital stay prior to LT ≥ 7 days, *n* (%)	20(74.1)		222(58.0)	0.100	
Infection within 2 months prior to LT, *n* (%)	17(63)		145(37.9)	0.010	
MELD score at LT ≥ 22, n (%)	21(77.8)		210(54.8)	0.020	
Pre-LT creatinine ≥ 2 mg/dL, *n* (%)	6(22.2)		23(6.0)	0.001	
Pre-LT WBC count ≥ 10 × 10^9^/L, *n* (%)	6(22.2)		53(13.8)	0.230	
Pre-LT lymphocyte count ≤ 0.5 × 10^9^/L, *n* (%)	9(33.3)		91(23.8)	0.263	
Pre-LT platelet count ≤ 50 × 10^9^/L, *n* (%)	8(29.6)		128(33.4)	0.686	
Pre-LT albumin level < 30 g/L, *n* (%)	6(22.2)		74(19.3)	0.713	
Donor age ≥ 50 years	6(22.2)		129(33.7)	0.221	
Steatosis ≥ 30%, *n* (%)	3(11.1)		39(10.2)	1.000	
Cold ischemia time ≥ 360 min, *n* (%)					
Duration of surgery ≥ 450 min, *n* (%)	7(25.9)		65(17)	0.237	
Intraoperative bleeding ≥ 3000 ml, *n* (%)	21(77.8)		217(56.7)	0.032	
Intraoperative RBC transfusion ≥ 12 U, *n* (%)	21(77.8)		202(52.7)	0.012	
ALT on day 1 after LT ≥ 1000U/L, *n* (%)	13(48.1)		120(31.3)	0.071	
Albumin level on day 1 after LT < 30 g/L, *n* (%)	3(11.1)		26(6.8)	0.647	
Creatinine on day 3 after LT ≥ 2 mg/dL, *n* (%)	16(59.3)		45(11.7)	< 0.001	
Post-LT FI, *n* (%)	9(33.3)		42(11.0)	0.001	
Acute rejection, *n* (%)	3(11.1)		64(16.7)	0.623	
Reoperation, *n* (%)	2(7.4)		15(3.9)	0.704	
Post-LT bacterial infection, *n* (%)	20(74.1)		100(26.1)	< 0.001	
Post-LT mechanical ventilation, *n* (%)	19(70.4)		76(19.8)	< 0.001	
Post-LT renal replacement therapy, *n* (%)	7(25.9)		12(3.1)	< 0.001	
Glucocorticoidse ≥ 1500 mg, *n* (%)	15(55.6)		243(63.4)	0.412	
Basiliximab use ≥ 40 mg, *n* (%)	7(25.9)		154(40.2)	0.142	
Multivariate analysis					
Recipient age ≥ 55 years				0.023	3.056(1.168–7.994)
Creatinine on day 3 after LT ≥ 2 mg/dL				< 0.001	9.154(3.451–24.280)
Post-LT bacterial infection				< 0.001	7.095(2.553–19.714)
Post-LT mechanical ventilation				< 0.001	5.742(2.176–15.151)

*ALT* alanine aminotransferase, *CI* confidence intervals, *FIs* fungal infections, *LT* liver transplant, *MELD* Model for end-stage liver disease, *OR* odds ratios, *RBC* red blood cell

Nonetheless, when performed another analysis of the factors related to prolonged ICU stay, multivariate logistic regression analysis identified FIs [OR = 3.027, 95%CI:1.558–5.878, *P* = 0.001] as an independent risk factor for ICU stay after LT ≥ 7 days, among other variables such as pre-LT creatinine ≥ 2 mg/dL [OR = 2.843, 95%CI:1.228–6.579, *P* = 0.015], re-operation [OR = 3.251, 95%CI:1.097–9.628, *P* = 0.033], post-LT bacterial infection [OR = 1.840, 95%CI:1.150–2.944, *P* = 0.011], and the need for post-LT mechanical ventilation [OR = 3.029, 95%CI:1.825–5.028, *P* < 0.001]. All information was summarized in Table 5.

**Table 5:** Univariate and multivariate Logistic regression analysis of risk factors for ICU stay after LT ≥ 7 days

Variables	ICU stay ≥ 7 d (152)	ICU stay < 7d (258)	*P*	OR (95% CI)
*Total, n (%)*				
Univariate analysis				
Male sex, *n* (%)	118(77.6)	220(85.3)	0.05	
Recipient age ≥ 55 years, *n* (%)	46(30.3)	56(21.7)	0.053	
Recipient BMI ≥ 25	38(25.0)	69(26.7)	0.698	
Viral cirrhosis/necrosis/tumor, *n* (%)	111(73.0)	193(74.8)	0.691	
Alcoholic cirrhosis, *n* (%)	11(7.2)	21(8.1)	0.742	
Pre-LT diabetes, *n* (%)	17(11.2)	31(12.0)	0.800	
Hospital stay prior to LT ≥ 7 days, *n* (%)	100(65.8)	142(55.0)	0.033	
Infection within 2 months prior to LT, *n* (%)	57(37.5)	105(40.7)	0.522	
MELD score at LT ≥ 22, *n* (%)	99(65.1)	132(51.2)	0.006	
Pre-LT creatinine ≥ 2 mg/dL, *n* (%)	18(11.8)	11(4.3)	0.004	
Pre-LT WBC count ≥ 10 × 10^9^/L, *n* (%)	27(17.8)	32(12.4)	0.135	
Pre-LT lymphocyte count ≤ 0.5 × 10^9^/L, *n* (%)	35(23.0)	65(25.2)	0.622	
Pre-LT platelet count ≤ 50 × 10^9^/L, *n* (%)	47(30.9)	89(34.5)	0.458	
Pre-LT albumin level < 30 g/L, *n* (%)	28(18.4)	52(20.2)	0.669	
Donor age ≥ 50 years	46(30.3)	89(34.5)	0.378	
Steatosis ≥ 30%, *n* (%)	16(10.5)	26(10.1)	0.885	
Cold ischemia time ≥ 360 min, *n* (%)				
Duration of surgery ≥ 450 min, *n* (%)	31(20.4)	41(15.9)	0.247	
Intraoperative bleeding ≥ 3000 ml, *n* (%)	102(67.1)	136(52.7)	0.004	
Intraoperative RBC transfusion ≥ 12 U, *n* (%)	93(61.2)	130(50.4)	0.034	
ALT on day 1 after LT ≥ 1000U/L, *n* (%)	56(36.8)	77(29.8)	0.144	
Albumin level on day 1 after LT < 30 g/L, *n* (%)	13(8.6)	16(6.2)	0.370	
Creatinine on day 3 after LT ≥ 2 mg/dL, *n* (%)	30(19.7)	31(12.0)	0.034	
Post-LT FIs, *n* (%)	34(22.4)	17(6.6)	< 0.001	
Acute rejection, *n* (%)	28(18.4)	39(15.1)	0.382	
Reoperation, *n* (%)	11(7.2)	6(2.3)	0.016	
Post-LT bacterial infection, *n* (%)	61(40.1)	59(22.9)	< 0.001	
Post-LT mechanical ventilation, *n* (%)	59(38.8)	36(14.0)	< 0.001	
Post-LT renal replacement therapy, *n* (%)	14(9.2)	5(1.9)	0.001	
Multivariate analysis				
Pre-LT creatinine ≥ 2 mg/dL			0.015	2.843(1.228–6.579)
Post-LT FIs			0.001	3.027(1.558–5.878)
Reoperation			0.033	3.251(1.097–9.628)
Post-LT bacterial infection			0.011	1.840(1.150–2.944)
Post-LT mechanical ventilation			< 0.001	3.029(1.825–5.028)

*ALT* alanine aminotransferase, *CI* confidence intervals, *FIs* invasive fungal infections, *LT* liver transplant, *MELD* Model for end-stage liver disease, *OR* odds ratios, *RBC* red blood cell

## Discussion

LT recipients are susceptible to opportunistic infections due to complex surgical procedures placing a greater number of patients at risk [26]. According to a recent study, half of the early fatal infections were mainly fungi-related [1]. The incidence of invasive FIs within 90 days after LT remains very high, reaching 12%, which was rather similar to our overall incidence of FIs (12.4%) which included all invasive and non-invasive FIs [1, 27–29] *Candida* represented the most frequently isolated species in LT recipients with invasive FIs in previous studies, also in line with our present cohort (84.3%) [1].

As previously reported, we observed that recipient age ≥ 55 years was independently associated with post-LT FIs. This goes along with recent findings by Breitkopf R, et al. that recipient age had an increased hazard ratio for breakthrough or whole invasive FIs within 90 days in a multivariate Cox regression model [2, 30].

We found that MELD score at LT ≥ 22 was association with the development of post-LT FIs, in accordance with the studies from in Saliba et al. and Utsumi et al. who concluded the association between high MELD score and post-LT invasive FIs [12, 19, 31].

We also established the association between pre-LT WBC count ≥ 10 × 10^9^/L and post-LT FIs. No previous studies reported elevated WBC influenced FIs development after LT. Hu et al. (2021) performed a meta analysis by combining data from 14 relevant studies enrolling 4,284 patients and concluded that bacterial infection was one of strong risk factors for invasive FIs after LT, which may partially explain why an elevated WBC count, in most cases, indicating the presence of an infection, was associated with FIs in our cohort [17].

As for intraoperative variables, our results suggest that blood loss ≥ 3000 ml was independently associated with post-LT FIs, in line with a previous study suggesting that excessive blood loss was one of risk factors for fungal infections in LT recipients [32].

We revealed post-LT duration of urethral catheter > 4 days as an independent risk factor for post-LT FIs. In a univariate analysis of bacterial and fungal infections after LT, Zhang W and colleagues found the association between urinary catheterization and infections; however, the significance did not remain in further multivariate analysis [33]. We should realize there is a reciprocal relationship between urinary catheterization and FIs. One the one hand, prolonged urinary catheterization may increase the risk for FIs; one the other hand, the FIs can also aggravate the disease and prolong the duration of urinary catheterization.

We confirmed that post-LT renal replacement therapy was correlated with FIs, consistent with a previous work [3]. A recent prospective multicenter study investigating the risk factors for breakthrough-invasive FI after LT also showed patients with and without breakthrough-invasive FI were significantly different for the need for renal replacement therapy after LT [29]. We confirmed that the risk for FIs was significantly reduced when a post-LT antifungal prophylaxis was implemented ≥ 3 d, in line with previous studies claiming that receipt of an antifungal agent was associated with decreased risk of post-LT invasive FIs after LT [12, 34–37].

Previous reports demonstrated that although most of mortality were unrelated to FIs, a high mortality was expected among immunocompromised patients with FIs [38, 39]. However, the effect of FIs on outcome of LT recipients is still a subject of discussion. Our present study revealed that FIs had a negative impact on ICU length of stay but had no impact on hospitalization stay after LT and 1-month all-cause mortality. Breitkopf R, et al. also confirmed the impact of invasive FIs on longer ICU stay and in contrast to our findings, they has linked invasive FIs and breakthrough invasive FIs with a significant impact on 1-year mortality [2, 30]. Now that the morbidity and mortality of invasive candidiasis and aspergillosis were high in LT recipients, targeted prophylaxis for *Candida spp.* and *Aspergillus spp.* was recommended since 2009 [40].

### Limits of the study

This study has several limitations. First, the retrospective single-center design implicates inherent selection bias and a limited generalizability, and only association rather than causation can be inferred. However, all consecutive LT recipients over a period of 8 years were investigated which represents one of the largest recently published studies in this field to examine the role of all FIs in outcomes as well as the risk factors for all FIs in an integrative manner. Second, the information of fungal colonization prior to LT would have provided insight into potential effects on the development of post-LT FIs which many studies stated, unfortunately these data were not available in our center [17]. Third, the heterogeneity of types of infection may lead to an inaccurate analysis of risk factors for IFs.

## Conclusion

All in all, although our study comprised a comparably large cohort of LT recipients, the burden of these infections on the fate of LT recipients emphasises the need for further prospective studies to identify these risk factors. Knowledge of these information aids in a successful LT as a result of prevention of FIs.

## Data sharing statement

The original anonymous dataset is available on request from the corresponding author at eraldo.occhetta@maggioreosp.novara.it.

## Data Availability

No datasets were generated or analyzed during the current study.

## Declarations

### STROBE statement

We have read the STROBE Statement—checklist of items, and the manuscript was prepared and revised according to the STROBE Statement—checklist of items.
